# Supplementary material for: Prevalence and determinants of late-stage presentation among cervical cancer patients, a systematic review and meta-analysis
Source: PLoS One. 2022 Apr 27;17(4):e0267571. doi: 10.1371/journal.pone.0267571 (PMC9045598; doi:10.1371/journal.pone.0267571)
Supplement: S1 File — (DOCX) [file pone.0267571.s002.docx]

Determinants of late-stage presentation among cervical cancer patients

|  | Population | Condition | Context |
| --- | --- | --- | --- |
|  | Cervical cancer Patients | Late stage presentation | World |
| Search terms: key words | Cervical cancer patient [tw] OR Cervical cancer cases [tw] | Late stage presentation [tw] OR late diagnosis[tw], delayed diagnosis[tw] OR advanced disease [tw] OR early diagnosis [tw] OR delayed presentation [tw] late tumour stage[tw] OR prolonged time to diagnosis [tw] delayed care seeking[tw] OR cancer presentation[tw] OR delayed access to care [tw] stage of diagnosis [tw] OR delayed treatment initiation[tw] OR patient delay [tw] OR delays in diagnosis [tw] OR stage at diagnosis [tw] OR advanced disease at presentation[tw] OR late-stage cervical cancer[tw] advanced stage at diagnosis [tw] |  |
|  |  |  |  |
|  |  |  |  |
|  |  |  |  |
|  |  |  |  |
|  |  |  |  |

1. Cervical cancer patient [tw] OR Cervical cancer cases [tw]
2. “Cervical cancer patient” [Mesh] OR “Cervical cancer cases” [Mesh]
3. Late stage presentation [tw] OR late diagnosis[tw], delayed diagnosis[tw] OR advanced disease [tw] OR early diagnosis [tw] OR delayed presentation [tw] late tumour stage[tw] OR prolonged time to diagnosis [tw] delayed care seeking[tw] OR cancer presentation[tw] OR delayed access to care [tw] stage of diagnosis [tw] OR delayed treatment initiation[tw] OR patient delay [tw] OR delays in diagnosis [tw] OR stage at diagnosis [tw] OR advanced disease at presentation[tw] OR late-stage cervical cancer[tw] advanced stage at diagnosis [tw]
4. “Late stage presentation” [Mesh] OR “late diagnosis” [Mesh] OR “delayed diagnosis” [Mesh] OR “advanced disease ” [Mesh] OR “early diagnosis ” [Mesh] OR “delayed presentation” [Mesh] OR “late tumour stage” [Mesh] OR “prolonged time to diagnosis” [Mesh] ‘”delayed care seeking “[Mesh] “ cancer presentation”[Mesh] OR “delayed access to care” [Mesh] OR “stage of diagnosis” [Mesh] OR “delayed treatment initiation” [Mesh] OR “patient delay” [Mesh] OR “delays in diagnosis” [Mesh] OR “stage at diagnosis” [Mesh] OR “advanced disease at presentation” [Mesh] OR “late-stage cervical cancer” [Mesh] OR “ advanced stage at diagnosis” [Mesh]
5. 1 OR 2
6. 3 OR 4
7. 5 AND 6
8. Limit 7 to Humans
9. Limit 7 to English

Search strategy

PubMed

Last searched on: July 20, 2021

| s/n | Query | Results | Time |
| --- | --- | --- | --- |
| 1 | Cervical cancer patient [tw] OR Cervical cancer cases [tw] | 877 | 02:41:52 |
| 2 | “Cervical cancer patient” [Mesh] OR “Cervical cancer cases” [Mesh] | 0 | 02:42:18 |
| 3 | Late stage presentation [tw] OR late diagnosis[tw], delayed diagnosis[tw] OR advanced disease [tw] OR early diagnosis [tw] OR delayed presentation [tw] late tumour stage[tw] OR prolonged time to diagnosis [tw] | 114 | 02:43:19 |
| 4 | “Late stage presentation” [Mesh] OR “late diagnosis” [Mesh] OR “delayed diagnosis” [Mesh] OR “advanced disease ” [Mesh] OR “early diagnosis ” [Mesh] OR “delayed presentation” [Mesh] OR “late tumour stage” [Mesh] OR “prolonged time to diagnosis” [Mesh] | 63,971 | 02:43:50 |
| 5 | 1 OR 2 | 877 | 02:45:30 |
| 6 | 3 OR 4 | 64,059 | 02:46:13 |
| 7 | 5 AND 6 | 83 | 02:47:09 |
| 8 | Limit 7 to Humans | 83 | 02:48:04 |
| 9 | Limit 7 to English | 81 | 03:48:22 |

Search number Query Sort By Filters Search Details Results Time

9 "((Cervical cancer patient [tw] OR Cervical cancer cases [tw]) OR (""Cervical cancer patient"" [Mesh] OR ""Cervical cancer cases"" [Mesh])) AND ((Late stage presentation [tw] OR late diagnosis[tw], delayed diagnosis[tw] OR advanced disease [tw] OR early diagnosis [tw] OR delayed presentation [tw] late tumour stage[tw] OR prolonged time to diagnosis [tw]) OR (""Late stage presentation"" [Mesh] OR ""late diagnosis"" [Mesh] OR ""delayed diagnosis"" [Mesh] OR ""advanced disease "" [Mesh] OR ""early diagnosis "" [Mesh] OR ""delayed presentation"" [Mesh] OR ""late tumour stage"" [Mesh] OR ""prolonged time to diagnosis"" [Mesh]))" "Humans, English" "((""cervical cancer patient""[Text Word] OR ""cervical cancer cases""[Text Word]) AND (((((""late stage presentation""[Text Word] OR ""late diagnosis""[Text Word]) AND ""delayed diagnosis""[Text Word]) OR ""advanced disease""[Text Word] OR ""early diagnosis""[Text Word] OR ""delayed presentation""[Text Word]) AND ""late tumour stage""[Text Word]) OR ((""prolong""[All Fields] OR ""prolongation""[All Fields] OR ""prolongations""[All Fields] OR ""prolonged""[All Fields] OR ""prolonging""[All Fields] OR ""prolongs""[All Fields]) AND ""time to diagnosis""[Text Word]) OR (""delayed diagnosis""[MeSH Terms] OR ""early diagnosis""[MeSH Terms]))) AND ((humans[Filter]) AND (english[Filter]))" 94 11:03:51

8 "((Cervical cancer patient [tw] OR Cervical cancer cases [tw]) OR (""Cervical cancer patient"" [Mesh] OR ""Cervical cancer cases"" [Mesh])) AND ((Late stage presentation [tw] OR late diagnosis[tw], delayed diagnosis[tw] OR advanced disease [tw] OR early diagnosis [tw] OR delayed presentation [tw] late tumour stage[tw] OR prolonged time to diagnosis [tw]) OR (""Late stage presentation"" [Mesh] OR ""late diagnosis"" [Mesh] OR ""delayed diagnosis"" [Mesh] OR ""advanced disease "" [Mesh] OR ""early diagnosis "" [Mesh] OR ""delayed presentation"" [Mesh] OR ""late tumour stage"" [Mesh] OR ""prolonged time to diagnosis"" [Mesh]))" Humans "((""cervical cancer patient""[Text Word] OR ""cervical cancer cases""[Text Word]) AND (((((""late stage presentation""[Text Word] OR ""late diagnosis""[Text Word]) AND ""delayed diagnosis""[Text Word]) OR ""advanced disease""[Text Word] OR ""early diagnosis""[Text Word] OR ""delayed presentation""[Text Word]) AND ""late tumour stage""[Text Word]) OR ((""prolong""[All Fields] OR ""prolongation""[All Fields] OR ""prolongations""[All Fields] OR ""prolonged""[All Fields] OR ""prolonging""[All Fields] OR ""prolongs""[All Fields]) AND ""time to diagnosis""[Text Word]) OR (""delayed diagnosis""[MeSH Terms] OR ""early diagnosis""[MeSH Terms]))) AND (humans[Filter])" 96 11:02:09

7 "((Cervical cancer patient [tw] OR Cervical cancer cases [tw]) OR (""Cervical cancer patient"" [Mesh] OR ""Cervical cancer cases"" [Mesh])) AND ((Late stage presentation [tw] OR late diagnosis[tw], delayed diagnosis[tw] OR advanced disease [tw] OR early diagnosis [tw] OR delayed presentation [tw] late tumour stage[tw] OR prolonged time to diagnosis [tw]) OR (""Late stage presentation"" [Mesh] OR ""late diagnosis"" [Mesh] OR ""delayed diagnosis"" [Mesh] OR ""advanced disease "" [Mesh] OR ""early diagnosis "" [Mesh] OR ""delayed presentation"" [Mesh] OR ""late tumour stage"" [Mesh] OR ""prolonged time to diagnosis"" [Mesh]))" "(""cervical cancer patient""[Text Word] OR ""cervical cancer cases""[Text Word]) AND (((((""late stage presentation""[Text Word] OR ""late diagnosis""[Text Word]) AND ""delayed diagnosis""[Text Word]) OR ""advanced disease""[Text Word] OR ""early diagnosis""[Text Word] OR ""delayed presentation""[Text Word]) AND ""late tumour stage""[Text Word]) OR ((""prolong""[All Fields] OR ""prolongation""[All Fields] OR ""prolongations""[All Fields] OR ""prolonged""[All Fields] OR ""prolonging""[All Fields] OR ""prolongs""[All Fields]) AND ""time to diagnosis""[Text Word]) OR (""delayed diagnosis""[MeSH Terms] OR ""early diagnosis""[MeSH Terms]))" 96 10:58:31

6 "(Late stage presentation [tw] OR late diagnosis[tw], delayed diagnosis[tw] OR advanced disease [tw] OR early diagnosis [tw] OR delayed presentation [tw] late tumour stage[tw] OR prolonged time to diagnosis [tw]) OR (""Late stage presentation"" [Mesh] OR ""late diagnosis"" [Mesh] OR ""delayed diagnosis"" [Mesh] OR ""advanced disease "" [Mesh] OR ""early diagnosis "" [Mesh] OR ""delayed presentation"" [Mesh] OR ""late tumour stage"" [Mesh] OR ""prolonged time to diagnosis"" [Mesh])" "((((""late stage presentation""[Text Word] OR ""late diagnosis""[Text Word]) AND ""delayed diagnosis""[Text Word]) OR ""advanced disease""[Text Word] OR ""early diagnosis""[Text Word] OR ""delayed presentation""[Text Word]) AND ""late tumour stage""[Text Word]) OR ((""prolong""[All Fields] OR ""prolongation""[All Fields] OR ""prolongations""[All Fields] OR ""prolonged""[All Fields] OR ""prolonging""[All Fields] OR ""prolongs""[All Fields]) AND ""time to diagnosis""[Text Word]) OR (""delayed diagnosis""[MeSH Terms] OR ""early diagnosis""[MeSH Terms])" "68,439" 10:57:44

5 "(Cervical cancer patient [tw] OR Cervical cancer cases [tw]) OR (""Cervical cancer patient"" [Mesh] OR ""Cervical cancer cases"" [Mesh])" """cervical cancer patient""[Text Word] OR ""cervical cancer cases""[Text Word]" 935 10:56:56

4 """Late stage presentation"" [Mesh] OR ""late diagnosis"" [Mesh] OR ""delayed diagnosis"" [Mesh] OR ""advanced disease "" [Mesh] OR ""early diagnosis "" [Mesh] OR ""delayed presentation"" [Mesh] OR ""late tumour stage"" [Mesh] OR ""prolonged time to diagnosis"" [Mesh]" """delayed diagnosis""[MeSH Terms] OR ""early diagnosis""[MeSH Terms]" "68,347" 10:56:03

3 "Late stage presentation [tw] OR late diagnosis[tw], delayed diagnosis[tw] OR advanced disease [tw] OR early diagnosis [tw] OR delayed presentation [tw] late tumour stage[tw] OR prolonged time to diagnosis [tw]" "((((""late stage presentation""[Text Word] OR ""late diagnosis""[Text Word]) AND ""delayed diagnosis""[Text Word]) OR ""advanced disease""[Text Word] OR ""early diagnosis""[Text Word] OR ""delayed presentation""[Text Word]) AND ""late tumour stage""[Text Word]) OR ((""prolong""[All Fields] OR ""prolongation""[All Fields] OR ""prolongations""[All Fields] OR ""prolonged""[All Fields] OR ""prolonging""[All Fields] OR ""prolongs""[All Fields]) AND ""time to diagnosis""[Text Word])" 120 10:55:24

2 """Cervical cancer patient"" [Mesh] OR ""Cervical cancer cases"" [Mesh] - Schema: all" """Cervical cancer patient"" [Mesh] OR ""Cervical cancer cases"" [Mesh]" 0 10:54:10

1 Cervical cancer patient [tw] OR Cervical cancer cases [tw] """cervical cancer patient""[Text Word] OR ""cervical cancer cases""[Text Word]" 935 10:53:05
