# Supplementary material for: Prevalence and determinants of late-stage presentation among cervical cancer patients, a systematic review and meta-analysis
Source: PLoS One. 2022 Apr 27;17(4):e0267571. doi: 10.1371/journal.pone.0267571 (PMC9045598; doi:10.1371/journal.pone.0267571)
Supplement: Attachment — Submitted filename: response to reviewers.docx [file pone.0267571.s005.docx]

REVIEWER COMMENTS

**Determinants of late-stage presentation among cervical cancer patients, a systematic
review and Meta-analysis**

Thank you for the opportunity to review this paper. This is an interesting topic but there so many issues that need to be addressed. First, the language is very poor. The authors need a support from a native English language writer or seek the journal’s language editing service. Second, the study objectives and methodology were poorly written. They stated under “outcome measure” that the primary measure was “prevalence” while the secondary measure was “determinants” but the title of this paper was “determinants”. So many missing information and not adhering to PRISMA guideline. Also not registered in PROSPERO. Results and tables were poorly presented. The authors should seek support from a librarian or an expert in systematic review to review this interesting paper. Below are a few comments. I could not present all errors I found in the paper due to its fundamental flaws.

Abstract:

1. The authors need to specify that they also want to estimate the prevalence and not only the determinants.

**Response** – corrected

2. Several descriptive and grammatical errors. Please have a review of the choice of word. Example first sentence in the results

*“From 64,059 obtained studies, 25 studies from 3 world regions involving 53,233 participants enrolled in this meta-analysis.”*

Also in the conclusion:

“…..*Therefore health care organizations should work on early screening and treatment, as well as increasing community awareness to minimize premature death among those patients is essential”* needs to be rephrased. The use of the term “therefore” doesn’t fit in.

Also see the Introduction section:

“*Several studies worldwide have investigated the factors associated with delayed diagnosis of the cancer and disparities in its mortality rate in different racial, geographic and socio-economic groups [10-13]. However knowledge of delays for this cancer could be useful in establishing comprehensive preventative strategies.”* The use of However is a problem here.

Please check other through out this paper. I found so many disconnect and wrong use of coordinating and subordinating conjunctions.

**Response** – corrected

Introduction

1. I would move this statement forward to the first or second paragraph:

“In Africa, high incidences of cervical cancer are reported at rates exceeding 50 per 100,000 populations [17]. In sub-Saharan Africa, cervical cancer is the second commonest cancer morbidity and the leading cause of mortality with over 577,000 deaths annually; furthermore, in Eastern Africa it is the number one commonest cancer in women [18].”

**Response** – corrected

2. “*Other factors were scattered studied so, this systematic
   review and meta-analysis aimed is to identify pooled determinants of late-stage presentation among cervical cancer patients”*???.

This sentence is not clear.

**Response** – corrected

3. Authors need to align their study objectives to the study title.

**Response** – corrected

Methods

1. The methodology of this review was poorly written. The authors claim to follow the PRISMA guideline, but I could not verify this in the methodology.

**Response** – corrected and followed updated PRISMA guideline

2. Looks like this systematic review was not registered in PROSPERO

**Response** – corrected and registration ID was mentioned

3. “…*The databases used were EMBASE, MEDLINE, Cochrane, Scopus, Web of Science,
   CINAHL, and manually on Google Scholar.”* This should be under information sources.

**Response** – corrected

4. “*Both funnel plot and Egger’s test had used to assess publication bias. A p-value< 0.05 had used to declare the statistical significance of publication bias. Also, I2 test statistics had used to check the heterogeneity of studies. I2 test statistics of < 50, 50–75% and > 75% was declared as low, moderate and high heterogeneity respectively [25]”.*

So many errors here and there and poorly written. The authors could not clearly describe their analytical approach and the reasons for what they did.

**Response** – corrected
